# Irradiation‐induced polyploid giant cancer cells are involved in tumor cell repopulation via neosis

**DOI:** 10.1002/1878-0261.12913

**Published:** 2021-02-17

**Authors:** Zhengxiang Zhang, Xiao Feng, Zheng Deng, Jin Cheng, Yiwei Wang, Minghui Zhao, Yucui Zhao, Sijia He, Qian Huang

**Affiliations:** ^1^ Cancer Center Shanghai General Hospital Shanghai Jiao Tong University School of Medicine Shanghai China; ^2^ Department of Oncology Henan Province People's Hospital Henan University Zhengzhou China

**Keywords:** HMGB1, irradiation, neosis, polyploid giant cancer cells, tumor repopulation

## Abstract

Tumor repopulation occurs when residual tumor cells surviving therapies tenaciously proliferate and re‐establish the tumor. The cellular and molecular mechanisms underlying this process remain poorly understood. In this study, we propose that polyploid giant cancer cells (PGCCs) are involved in tumor repopulation via neosis following radiotherapy. We found that although the majority of PGCCs induced by irradiation underwent cell death, some PGCCs exhibited proliferative capacity. Utilizing time‐lapse microscopy and single‐cell cloning assays, we observed that proliferating PGCCs underwent neosis, thereby contributing to tumor cell repopulation after irradiation. Notably, HMGB1 released from dying tumor cells rather than intracellular HMGB1 could promote neosis‐based tumor repopulation, and the latter could be suppressed by the use of HMGB1 inhibitors. Taken together, our results indicate that PGCC can initiate tumor repopulation via neosis following radiation therapy.

## Graphical abstract

In this study, we propose that polyploid giant cancer cells (PGCCs) are involved in tumor repopulation following radiotherapy. Although the majority of PGCCs induced by irradiation underwent cell death, some PGCCs exhibited proliferative capacity, identified as neosis. HMGB1 released from dying tumor cells rather than intracellular HMGB1 could promote neosis‐based tumor repopulation.

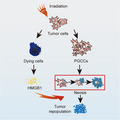

**Abbreviations**
DAPI: 4’,6‐diamidino‐2‐phenylindole
DAMP: damage associated molecular pattern
DMEM: Dulbecco’s modified Eagle’s medium
eIF4E: eukaryotic initiation factor 4E
EP: ethyl pyruvate
FACS: fluorescence activating cell sorter
GL: glycyrrhizinate
HMGB1: high mobility group box 1 protein
IHC: immunohistochemical staining
PGCCs: polyploid giant cancer cells
PI: propidium iodide
PKCδ: protein kinase Cδ
PVDF: polyvinylidene difluoride membrane
RIPA: radioimmunoprecipitation
RCs: Raju cells
RAGE: receptor for advanced glycation endproducts
Sox2: sex‐determining region Y‐box 2
TUNEL: terminal deoxyribonucleotidyl transferase‐mediated dUTP nick end labeling

## 1 Introduction

Tumor repopulation, a great predicament in cancer therapeutics, generally denotes that surviving tumor cells undergoing therapies (e.g. radiotherapy, chemotherapy) continuously proliferate and unfavorably re‐establish the tumor. It is conceivable that unfolding the mechanisms responsible for tumor repopulation may bring novel therapeutic strategies to restrict tumor repopulation and improve clinical outcomes. Emerging studies have focused on unraveling the mechanisms underlying tumor repopulation after therapy. Huang *et al*. [1] demonstrated that radiotherapy‐induced dying tumor cells exerted great potential to stimulate surrounding tumor cell proliferation through a Caspase‐3‐dependent mechanism. Another successive study recapitulated that Caspase‐3 in cytotoxic therapy‐induced dying melanoma cells prompted melanoma repopulation after therapy [2]. In addition, our laboratory revealed that PKCδ, which can be cleaved and activated by cleaved Caspase‐3, mediated pancreatic tumor cell repopulation as a downstream factor of Caspase‐3 [3, 4]. Besides Caspase‐3‐centered tumor repopulation mechanisms, one recent study from our laboratory uncovered that eIF4E‐phosphorylation‐mediated sex‐determining region Y‐box 2 (Sox2) upregulation facilitates pancreatic tumor cell repopulation following irradiation [5]. Though these studies have enriched theories and molecular mechanisms of tumor repopulation, understanding the observably morphological mechanisms at cellular level, which may directly display the process of tumor repopulation, remain elusive.

Polyploid giant cancer cells (PGCCs) are ubiquitous in human cancerous tissues, which contain enlarged or multiple nuclei with increased genomic content when compared with other cancer cells in the same tumor [6, 7, 8, 9]. The formation of PGCCs can be induced by hypoxia, chemotherapeutic drugs and irradiation [6, 10, 11, 12]. The main mechanisms responsible for the formation of PGCCs are associated with cell fusion [13], endoreplication [14, 15], cytokinesis failure[14, 15] and cell cannibalism by entosis [16], eventually contributing to generation of polyploid cells. It is well documented that PGCCs are associated with resistance to cancer therapy [7, 17, 18, 19]. Targeting PGCCs may provide a new therapeutic approach in cancer therapy.

Neosis, a novel manner of cell division, was first reported by Sundaram *et al*. [20]. Before the demise of PGCCs, some of them can undergo neosis characterized by karyokinesis via nuclear budding, asymmetrically intracellular cytokinesis and production of small mononuclear cells (termed Raju cells) [20]. The newly formed Raju cells via neosis were considered to play a role in self‐renewal in cancer [21, 22], therapeutic resistance [23, 24] due to their stem‐like traits [25, 26, 27]. Therefore, revealing the molecular events whereby PGCC‐derived neosis promotes tumor recurrence or repopulation after therapy can lead to clinically relevant approaches for preventing tumor relapse.

In this study, we found that the PGCCs induced by irradiation could move towards both demise and survival. Importantly, using single‐cell cloning assay, we observed that those PGCCs with potentially proliferative capacity eventually promoted tumor cell repopulation via neosis. In a further step, we demonstrated that HMGB1 secreted by dying tumor cells promoted neosis in a paracrine manner. We hope that this study can at cellular and molecular levels provide a novel perspective on the role of PGCC‐derived neosis in tumor repopulation after radiotherapy.

## 2 Materials and methods

### 2.1 Cell culture and irradiation

The breast cancer cell line 4T1, MDA‐MB‐231 cells and human cervical carcinoma cell line HeLa cells were purchased from the Chinese Academy of Science (Shanghai, China) and cultured in Dulbecco’s modified Eagle’s medium (DMEM) (Thermo Scientific Inc., Beijing, China) containing 10% FBS (Tianhang Biological Technology Co., Ltd, Hangzhou, China), supplemented with 1% penicillin and 1% streptomycin at 37 °C under 5% CO_2_. Cells were irradiated with X‐rays using an Oncor linear accelerator (Siemens, Amberg, Germany). The dose rate was 3.6 Gy·min^−1^.

### 2.2 Clonogenic cell survival assay

4T1 cells growing in 6‐cm dishes with different cell density were irradiated with various doses of X‐ray (400, 600, 1000, 2000, 10 000 and 1 00 000 cells with 0Gy, 2Gy, 4Gy, 6Gy, 8Gy and 10Gy, respectively). After 14‐day culture at 37 °C in 5% CO_2_ incubator, colonies were fixed for 15 min with methanol and stained with crystal violet in 50% methanol. The number of colonies was counted with a cut‐off of 50 viable cells. The surviving fraction was then calculated as described in published instructions [28]. To test the ability of PGCCs to form a colony, we collected irradiation‐induced PGCCs and plated the cells into 6‐cm dishes with cell density of 20 000 cells. The rest process was the same as before [28].

### 2.3 Dead cell counting by flow cytometry

Exponentially growing cells were seeded into 6‐cm dishes, irradiated 48 h later, trypsinized at the specific time points, and stained with Annexin V and propidium iodide (PI) using FITC Annexin V Apoptosis Detection Kit from Becton Dickinson (BD). Apoptosis was measured by Accuri C6 Flow Cytometer from BD.

### 2.4 Cell cycle analysis by FACS

The cells in 10‐cm dishes were trypsinized, washed and fixed with 70% ethanol at the indicated time points and stored at −20 °C for at least 12 h. Cells were then washed and incubated with PI/RNase staining buffer (BD Pharmingen) for 15 min at room temperature. The cells were analyzed on the Accuri C6 flow cytometer. The modfit lt software (Verity Software House, Topsham, ME, USA) was used to calculate fitted cell cycle values.

### 2.5 Western blot

Cell extracts were prepared in RIPA lysis buffer supplemented with protease inhibitor cocktail (Roche, Basel, Switzerland). Samples mixed in loading buffer (Beyotime, Jiangsu, China) were heated to 100 °C for 10 min, separated on SDS‐polyacrylamide gel by electrophoresis, and electrotransferred onto polyvinylidene difluoride membrane (PVDF) membrane (Bio‐Rad, Hercules, CA, USA). The membranes were incubated with primary antibodies overnight at 4 °C and then with secondary antibodies for 2 h at room temperature. ECL Plus (Roche) was used to visualize the signals on the membrane. To obtain cell extracts that we needed, we irradiated cancer cells with 10Gy and collected the mixture after 5 days, when amount of PGCCs occurred according to our previous observation, and named it ‘PGCC + RC’. We then collected cells extracts after 10 days, when Raju cells proliferated to form a clone, and named it ‘RC’.

### 2.6 Antibodies and inhibitors used in this study

Antibodies (PCNA, Caspase‐3, Cleaved Caspase‐3, Cyclin D1, γ‐H2AX, p‐P38, p‐ERK, Sox2) were purchased from Cell Signaling Technology (Beverly, MA, USA). Antibodies (HMGB1, RAGE) were purchased from Abcam (Burlingame, CA, USA). Quantification of signals was performed using Image Quant TL (Amersham Bioscience, Marlborough, MA, USA). Ethyl pyruvate (EP) and glycyrrhizinate (GL) were purchased from Sigma‐Aldrich (St. Louis, MO, USA).

### 2.7 Immunocytochemical and immunofluorescence staining

Cells grown on growth coverglasses (Fisher Scientific, Pittsburgh, PA, USA) positioned on the bottom of 24‐well dishes or specialized confocal dishes with a coverglass bottom (In Vitro Scientific, CA, USA), were irradiated 48 h after seeding. At the indicated time points, the cells were fixed in paraformaldehyde (4% in PBS, pH 7.4), permeabilized with 0.3% Triton X‐100, and blocked with 5% donkey serum in PBS for 1 h at room temperature. The cells were then incubated with primary antibodies at 4 °C overnight, followed by incubation with fluorochrome‐labeled secondary antibodies (Biotium, Hayward, CA, USA) for 20 min at room temperature. For double immunofluorescence, primary antibodies raised in a mixture of rabbit and mouse species, and a mixture of CF633‐labeled anti‐rabbit and CF488A‐labeled anti‐mouse secondary antibodies (Biotium) were used. Nuclei were counterstained with DAPI (Beyotime, Jiangsu, China) and cells were analyzed using a Leica SP8 confocal laser scanning microscope. For immunocytochemistry, the GtvisionIII detection system (Gene Tech, Shanghai, China) was used as an equivalent of secondary antibody after incubation of primary antibody as previously shown. Substrate diaminobenzidine was then added to visualize positive immune reaction. Nuclei were counterstained with hematoxylin.

### 2.8 Terminal deoxyribonucleotidyl transferase‐mediated dUTP nick end labeling

Cells grown on cover glasses were fixed in paraformaldehyde (4% in PBS, pH 7.4), permeabilized (0.3% Triton X‐100 in PBS), and then incubated in the terminal deoxyribonucleotidyl transferase‐mediated dUTP nick end labeling (TUNEL) reaction mixture (In Situ Cell Death Detection Kit, TMR red, from Roche, America) for 1 h. The TUNEL‐positive cells were finally analyzed using Leica SP8 confocal laser scanning microscope.

### 2.9 Transmission electron microscopy

Exponentially growing cells in 10‐cm dishes were irradiated with 10Gy doses. After 4 days, the adherent cells were fixed in 2.5% glutaraldehyde solution overnight and then scraped off with cell scraper. After centrifugation and washing with PBS, the cells was fixed with osmium tetroxide solution for at least 2 h, and dehydrated with gradient alcohol and propylene oxide. The cell pellet was exposed to a mixture of propylene oxide/resin for infiltration and then for embedding. Sectioning was accomplished with the use of an LKB Ultratome V (LKB‐Produkter, Bromma, Sweden). The thin sections were transferred into a copper grid and then stained with lead citrate for viewing under a Philips CM‐120 transmission electron microscope (Philips, Amsterdam, Netherlands).

### 2.10 BrdU immunofluorescence staining and BrdU chasing assay

A final concentration of 0.03 mg·mL^−1^ of BrdU (Sigma‐Aldrich) in pre‐warmed growth medium was added to cells grown on coverslips and incubated at 37 °C for 30 min. The cells were fixed in paraformaldehyde (4% in PBS, pH 7.4) and then incubated in 1.5 m HCl for 30 min at room temperature to denature DNA. BrdU primary antibody (Cell Signaling Technology) was then used to detect the incorporated BrdU in cells through immunofluorescence staining as shown above.

BrdU pulse‐chase assay was performed to track BrdU‐labeled PGCCs. BrdU (0.03 mg·mL^−1^) was added to cells for one pulse incubation for 6 h to make the most BrdU‐labeled cells after the cells were irradiated. At 6 h, 2 days, 5 days, 10 days and 15 days after labeling, remnant‐incorporated BrdU was detected by immunofluorescence staining.

### 2.11 Time‐lapse observation of 4T1 PGCCs following irradiation

4T1 cells seeded in a 24‐well plate at a density of 20 000 per well were irradiated with 10Gy doses. The morphological change of PGCCs was analyzed by the IncuCyte ZOOM Live‐Cell Analysis System (Essen, Wilmington, DE, USA), two sampling points were selected from every well of the plate and photographed every 2 h for 5 days.

### 2.12 The record of the PGCCs and neosis and single‐cell cloning assay

The irradiated 4T1 cells were cultured for 4 or 5 days until the largest numbers of PGCCs appeared. The cells were then trypsinized and 1000 cells were seeded into 10‐cm Petri dishes for further culture. On the following day, the single adherent viable cells were labeled and photographed every 12 h under a phase contrast microscope. To confirm that a signal tumor cell treated as above was able to form clones from PGCC by neosis, a signal tumor cell treated as above was plated into a signal well of a 96‐well plate by FACS sorting. From the next day, the well with adherent cells was marked and observed for 14 days. The plate was stain with crystal violet and the colony with > 50 cells was counted.

### 2.13 Hoechst 33342 and PI staining assay

For apoptosis determination, Hoechst 33342/PI double stain kit (Solarbio, Beijing, China) was used according to instructions. Briefly, cells were washed with PBS and exposed to Hoechst 33342 (5 μL) and PI (5 μL) for 20 min at 4 °C in the dark, and then washed again with PBS. A fluorescence microscope (Leica DM4000B, Germany) was used to examine Hoechst 33342 and PI fluorescence staining in each cell.

### 2.14 Soft agar colony formation assay

The soft agar colony formation assay was carried out as previously described [29]. Briefly, we coated the bottom of 6‐well plates with 0.6% agar solution, which was dissolved in complete medium. After solidifying, 0.35% agar solution containing PGCCs was added at the top and incubated in a humidified atmosphere of 5% CO_2_ for 10 days. Colonies were stained with crystal violet and captured using a Leica microscope.

### 2.15 Animal models

All of the BALB/c nude mice were brought from the Shanghai SLAC Laboratory Animal Co. Ltd (Shanghai, China). In the animal tumor‐bearing model, 1 × 10^6^ cells suspended in 100 μL PBS were subcutaneously injected into the hind leg of 6‐week‐old BALB/c nude mouse. When tumor volume reached 200 m^3^, the nude mice were subjected to 10Gy partial irradiation centralized on the tumor. Tumor volume was measured twice a week using the formula: *V* = (length × width^2^)/2. All animal experiments were performed according to The Animal Care and Use Committee of Shanghai Jiao Tong University School of Medicine.

### 2.16 HE staining

The formalin‐fixed, paraffin‐embedded spheroid tissue was cut into 4‐µm sections, deparaffinized and rehydrated. The tissue sections were then counterstained with hematoxylin for 1 min and eosin for 2 min, dehydrated and mounted on coverslips.

### 2.17 Immunohistochemical staining

The 4‐µm sections were washed with PBS and then subjected to antigen retrieval in 0.01 m sodium citrate buffer, heated in a water bath for 10 min. The sections were incubated with primary antibodies at 4 ℃ overnight in a humidifying chamber. Streptavidin biotin system was used to detect the signal in the presence of the chromogen 3,3’‐diaminobenzidine or alkaline phosphatase.

### 2.18 Statistical analysis

Student’s two‐sided test was used to compare the significance between control and treated groups. Multiple group comparisons were performed using one‐way analysis of variance (one‐way ANOVA). *P* < 0.05 was considered statistically significant.

## 3 Results

### 3.1 Fate of irradiated tumor cells

**Fig. 1:**
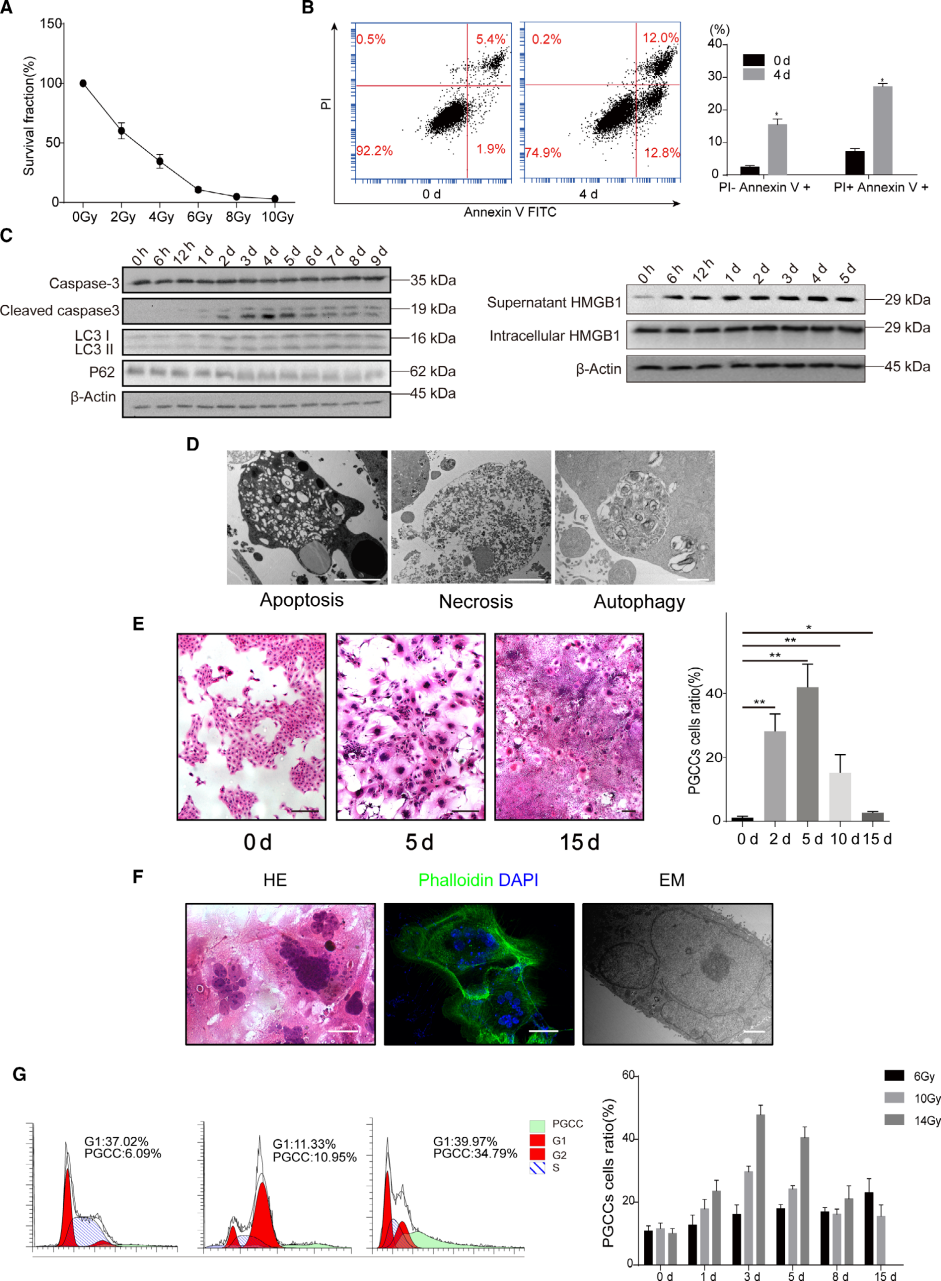
Irradiation induced the formation PGCCs. (A) Clonogenic survival assay showing the survival fraction of 4T1 cells under various doses of irradiation. (B) Flow cytometry assay detecting manner of cell death in 4T1 cells 4 days after 10Gy irradiation. (C) Western blot analysis showing increase of Cleaved Caspase‐3 and secreted HMGB1 in supernatant and decrease of P62 in 4T1 cells after 10Gy irradiation. (D) Transmission electron microscopy showing morphological traits of apoptosis, necrosis and autophagy. Scale bar: 5 μm. (E) Left panel, representative pictures showing PGCC formation in 4T1 cells after 10Gy irradiation over time. Scale bar: 200 μm. Right panel, quantitative analysis showing the change of ratios of PGCCs in 4T1 cells after 10Gy irradiation over time. (F) Left panel, HE staining showing the enlarged PGCCs. Middle panel, Phalloidin staining showing PGCCs. Right panel, transmission electron microscopy showing PGCCs. Scale bar: 50 μm. (G) Left panel, flow cytometry detecting cell cycle revealed that the percentage of polyploid cells in 4T1 cells increased significantly 3 days after 10Gy irradiation. Right panel, plot showing the ratios of PGCCs in 4T1 cell under different doses of irradiation over time. *n* = 3, data are shown as mean ± SD. Student’s *t*‐test was used to determine statistical significance: *P < 0.05, **P < 0.01. [Colour figure can be viewed at wileyonlinelibrary.com]

We first determined the influence of irradiation on tumor cell fate. Clonogenic cell survival assay showed the survival fraction of 4T1 cells in response to different doses of X‐ray irradiation (Fig. 1A). For example, 10Gy irradiation killed most of 4T1 cells; only approximately 1.6% cells survived and formed colonies. Flow cytometry analysis relying on FITC Annexin V and PI double staining showed that the percentage of 4T1 cells in early apoptosis and necrosis increased significantly 4 days after 10Gy irradiation (Fig. 1B). Western blot analysis manifested that the expression of Cleaved Caspase‐3, secreted HMGB1 and ratio of LC3II/I rose after 10Gy irradiation, indicating that various manners cell death existed after irradiation, such as apoptosis, necrosis and autophagy (Fig. 1C). Transmission electron microscopy confirmed these different kinds of cell death (Fig. 1D).

### 3.2 Irradiation induces the formation of PGCCs

Despite the different kinds of cell death after irradiation, we also observed morphological change of these cells. Intriguingly, we found that the cell size of some 4T1 cells after irradiation continuously increased and hundreds of multinuclei and micronuclei could be seen in even a single enlarged cell. These cells were named PGCCs (Fig. 1E). PGCCs existed after irradiation, and the percentage of these PGCCs grew rapidly in the following days, reaching peak value (42%) at day 5 and decreasing in the following days (Fig. 1E). To confirm the existence of PGCCs after irradiation, immunofluorescence and electron microscopy analysis were utilized, with DAPI for nucleus and phalloidin for cytoskeleton. The cytoskeleton staining by phalloidin indicated that multiple nuclei were located inside a single PGCC (Fig. 1F). PGCC could also be clearly seen in the transmission electron microscope (Fig. 1F). Cell cycle analysis, which identified PGCCs as polyploid cells, was done further to figure out PGCC changes over time following irradiation under different doses of irradiation. Cell cycle analysis revealed that the percentage of PGCCs (polyploid 4T1 cells) was greatly augmented after 10Gy irradiation, ranging from 6.09% to 34.79% (Fig. 1G). The higher the radiation dose, the more PGCCs (Fig. 1G). These results revealed the multinucleated trait of PGCCs and excluded the possibility that PGCCs were an aggregate of many cells or cellular debris after irradiation. The PGCCs induced by irradiation were characterized by significantly increased cell size, multiple nuclei or giant nuclei gathered in the center of cell, with the micronuclei widely scattered around the multiple nuclei. The formation of PGCCs existed not only during irradiation but also after chemotherapy (Fig. S1), which is consistent with previous reports [6, 18, 26].

### 3.3 PGCCs formed after irradiation exhibited proliferation potential

**Fig. 2:**
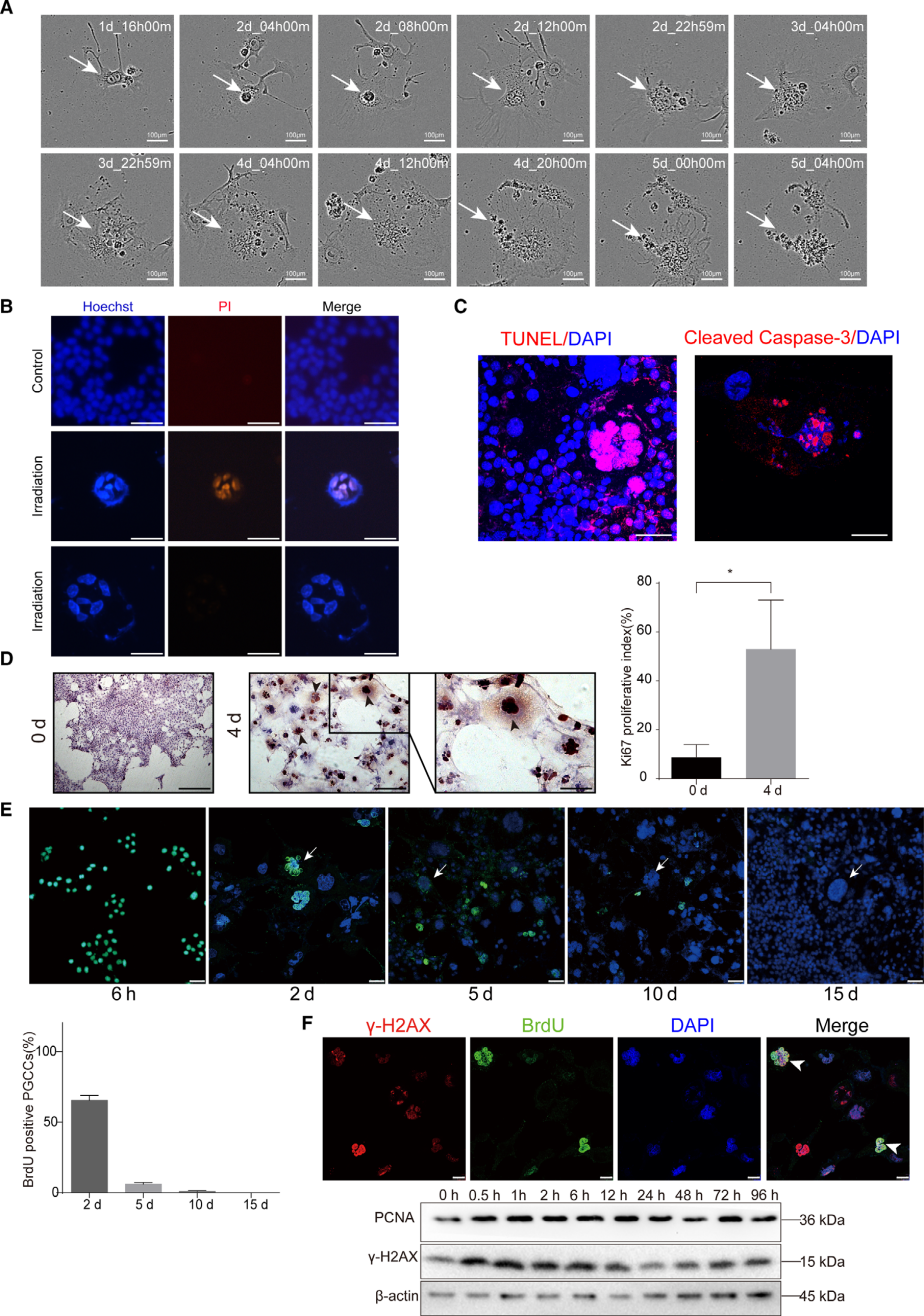
Irradiation‐induced PGCCs possessed proliferation potential. (A) Time‐lapse microscopy monitored the demise process of PGCCs. (B) Hoechst 33342/PI double staining showed that irradiation‐induced PGCCs underwent apoptosis. Scale bar: 100 μm. (C) TUNEL and Cleaved Caspase‐3 staining showing positive staining for some PGCCs. Scale bar: 50 μm. (D) Left panel, representative picture showing Ki67 staining for non‐irradiation cells and PGCCs. (Left panel, Ki67 staining in non‐irradiation cells. Scale bar: 250 μm. Middle panel, Ki67 staining in irradiation‐induced PGCCs. Scale bar: 100 μm. Right panel, a high‐magnification image to show positivity of Ki67 staining in PGCCs. Scale bar: 50 μm. Right panel, quantitative analysis showing the Ki67 proliferative index. (E) BrdU pulse‐chase assay to track BrdU‐labeled PGCC cells. Arrows indicated PGCCs. Scale bar: 50 μm. Lower panel, quantitative analysis showing the BrdU positive PGCCs percentage. (F) Upper panel, immunofluorescence staining showing irradiation induced PGCCs proliferated in spite of DNA damage. Lower panel, western blot analysis showing the PCNA, marker of proliferation, and γ‐H2AX, marker of DNA damages increased synchronously after 10Gy irradiation over time. Scale bar: 25 μm. *n* = 5, data are shown as mean ± SD. Student’s *t*‐test was used to determine statistical significance: *P < 0.05, **P < 0.01. [Colour figure can be viewed at wileyonlinelibrary.com]

**Fig. 3:**
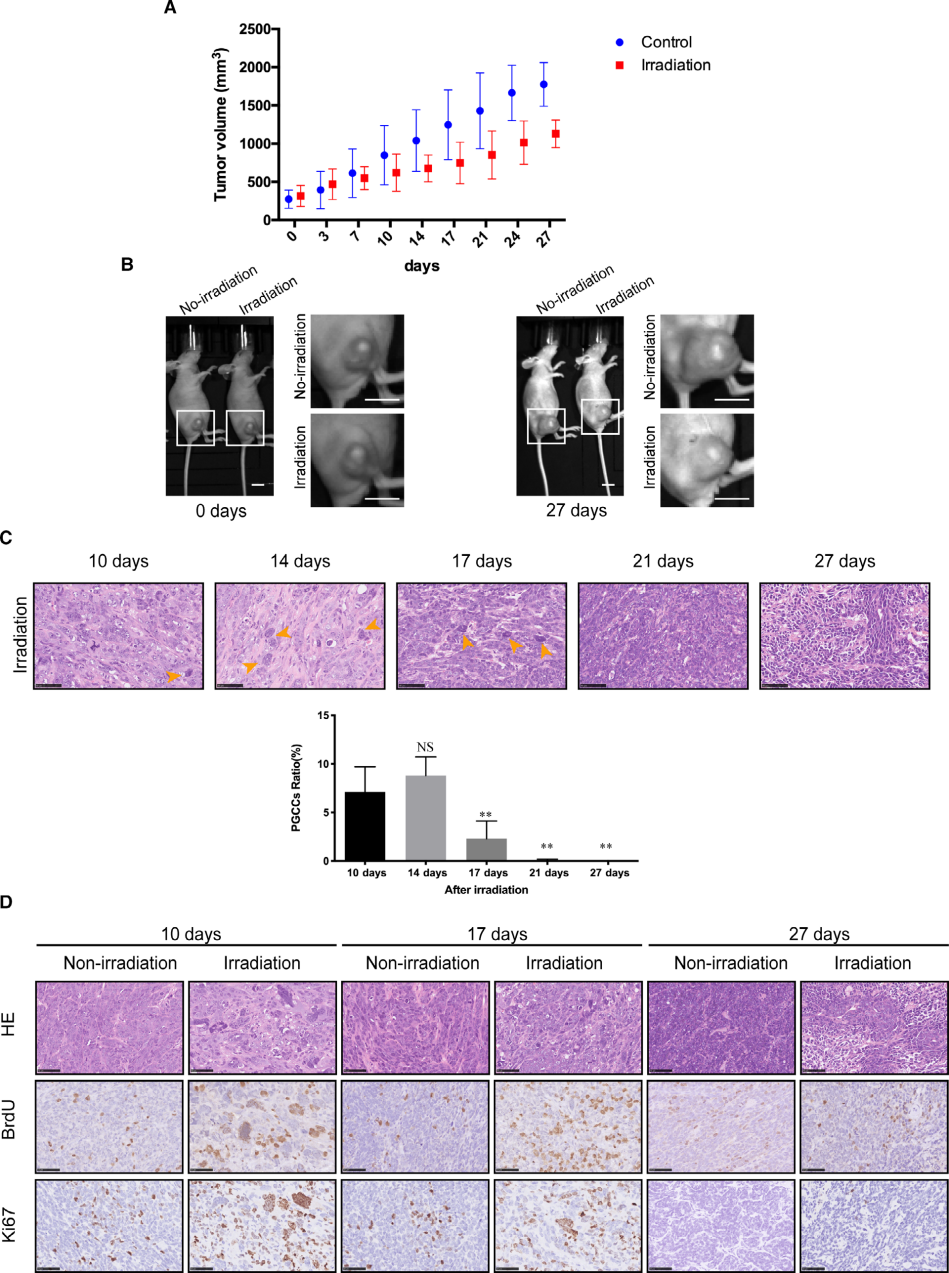
Irradiation induced the formation of proliferative PGCCs *in vivo*. (A) A graph of the tumor volume in non‐irradiation (control) and irradiation group. (B) The photograph of tumor volume captured by IVIS Lumina Series III imaging machine. Scale bar: 1 cm. (C) Upper panel, HE staining showed the formation of PGCCs after irradiation. Orange arrow indicated PGCCs. Scale bar: 50 μm. Lower panel, histogram of PGCC cells ratio according to HE staining. (D) Immunohistochemical staining of BrdU and Ki67 in non‐irradiation and irradiation group, in which some PGCCs stained positive with BrdU and Ki67. Scale bar: 50 μm. *n* = 5, data are shown as mean ± SD. Student’s *t*‐test was used to determine statistical significance: *P < 0.05, **P < 0.01. [Colour figure can be viewed at wileyonlinelibrary.com]

To determine the destiny of PGCCs induced by irradiation, real‐time live‐cell tracing was performed to record the death course of a single PGCC after 10Gy X‐ray irradiation using the Incucyte ZOOM Live Cell Imager. The results showed that PGCCs gradually began to die at day 3, and were fully disintegrated at day 5 (Fig. 2A). We utilized Hoechst 33342/PI double staining to show that some of PGCCs underwent apoptosis (PI staining positive), whereas others did not (Fig. 2B). The apoptosis of PGCCs was also detected with TUNEL and immunofluorescence for Cleaved Caspase‐3 (Fig. 2C). Since some of these PGCCs were not apoptotic (Fig. 2B), we performed the staining of proliferative markers Ki67. Compared with normal non‐irradiated cells, the Ki67 index was obviously increased 4 days after irradiation. Moreover, Ki67 was demonstrated to be strongly positive in most of PGCCs (Fig. 2D). To investigate whether PGCCs had mitotic capacity, BrdU pulse‐chase assay was performed. Full dose BrdU was administered to 4T1 cells immediately after X‐ray irradiation so the majority of the cells were able to incorporate exogenous BrdU. The content and distribution of the remaining BrdU were then tracked. Although almost all the cells were BrdU‐positive at 6 h, cells staining BrdU‐positive decreased sharply in the next few days, and the green fluorescence of BrdU was not observed in the newly formed PGCCs (Fig. 3E). The immunofluorescence colocalized the signals of BrdU and γ‐H2AX, showing that the proliferative PGCCs indeed experienced DNA double strand injury imposed by irradiation (Fig. 2F). Western blot confirmed that PCNA, a well‐known proliferative marker, rose with increased γ‐H2AX, a cell damage signal, 30 min after irradiation, indicating that increased proliferative activity with ionizing damage is a rapid reaction (Fig. 2F).

To confirm PGCC existence after irradiation *in vivo*, we utilized 10Gy irradiation to treat the animal tumor‐bearing model. Irradiation treatment slowed down the rate of tumor growth (Fig. 3A,B). HE staining was used to detect the morphological change of these irradiated tumor cells. Compared with the non‐irradiation group, PGCCs were observed in the irradiation group after 10 days, and increased in the following 4 days. After 21 days, PGCCs were rarely seen in tumor tissue and most tumor cells returned to the normal size of the non‐irradiation group (Fig. 3C). To test whether these PGCCs also presented proliferation potential as the *in vitro* assay, BrdU and Ki67 immunohistochemical staining were applied. Some PGCCs were Ki67‐ and BrdU‐positive, indicating that these PGCCs had proliferation potential (Fig. 3D).

### 3.4 PGCCs were involved in tumor repopulation via neosis

**Fig. 4:**
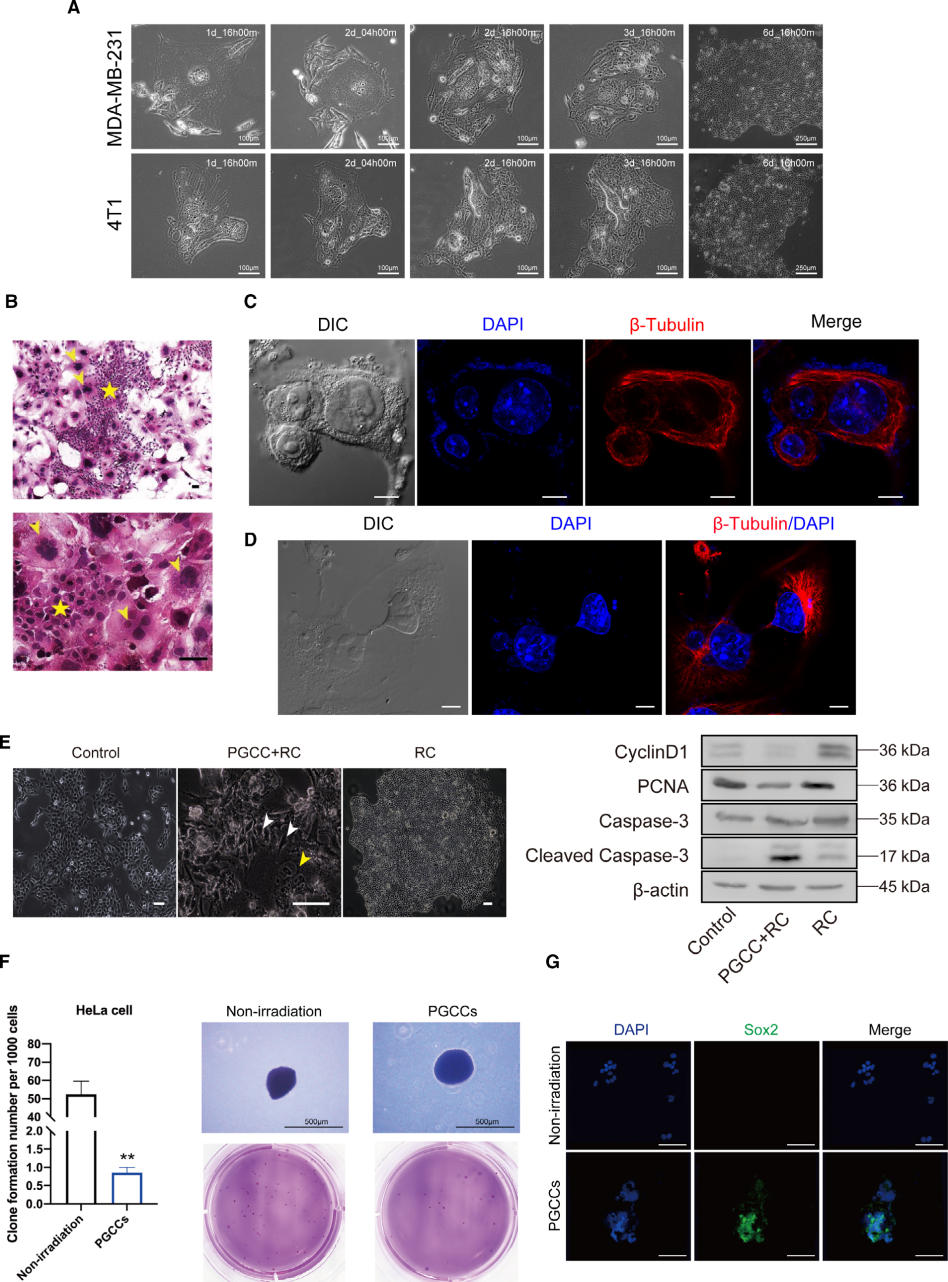
Polyploid giant cancer cells contribute to tumor repopulation via neosis. (A) Time‐lapse microscopy monitored the process of neosis from a single PGCC. (B) Representative pictures showing PGCCs and Raju cells in 4T1 cells after 10Gy irradiation. Yellow arrow indicated PGCCs and yellow pentagram indicates Raju cells. Scale bar: 50 μm. (C) Cytoskeleton β‐tubulin immunofluorescence showed that the nucleoplasmic bridge linked nucleus of the two daughter cells derived from one PGCC. Scale bar: 10 μm. (D) β‐tubulin staining showing the process of neosis, in which two small Raju cells are being expelled from a single PGCC. Scale bar: 10 μm. (E) Left panel, Representative pictures showing different populations of 4T1 cells. White arrow indicates Raju cells (RC) and yellow arrow indicated PGCCs. Right panel, western blot analysis showing difference in cell cycle, proliferation and apoptosis among three populations of cells. (F) Soft agar assay showing the different spheroid formation ability of non‐irradiation cells and PGCCs. Right panel, quantitative analysis showing the clone formation number per 1000 cells. Left panel, the image of clone formed in soft agar. Scale bar: 500 μm. (G) Immunofluorescent staining of Sox2 in non‐irradiation cells and PGCCs. Scale bar: 500 μm. *n* = 3, data are shown as mean ± SD. Student’s *t*‐test was used to determine statistical significance: *P < 0.05, **P < 0.01. [Colour figure can be viewed at wileyonlinelibrary.com]

In this study, we found that some PGCCs were proliferative with mitotic activity. To determine whether these PGCCs had the proliferative potential to form a new tumor, we used single‐cell cloning assay to observe the fate of PGCCs. Through time‐lapse observation, we found that some PGCCs in the 96‐well plate could generate numerous small mononuclear cells (Raju cells, RCs) and eventually form cell colonies, a process termed neosis (Fig. 4A). HE staining verified the formation of PGCCs and RCs after irradiation (Fig. 4B). These RCs might come from PGCC budding or cell division (Fig. 4C,D). We speculated that these new cells formed through neosis were responsible for tumor cell repopulation. Using western blot analysis, we detected the proliferative ability of RCs. Results showed that RCs expressed higher levels of Cyclin D1 and PCNA compared with non‐irradiated 4T1 cells and a lower level of Cleaved Caspase‐3, compared with the mixture of PGCCs and RCs (Fig. 4E), indicating that RCs had a greater proliferative ability and a lower level of apoptosis. Soft agar assay and immunofluorescence staining of stem cell marker Sox2 were performed to verify the repopulation and stemness potential of PGCCs, respectively. As shown in Fig. 4, these irradiation‐induced PGCCs had the potential to form a clone (Fig. 4F) and showed Sox2‐positive staining (Fig. 4G), which indicated that PGCCs had long‐term clonogenic and stemness potential.

### 3.5 HMGB1 released from dying cells mediated neosis‐initiating tumor repopulation

**Fig. 5:**
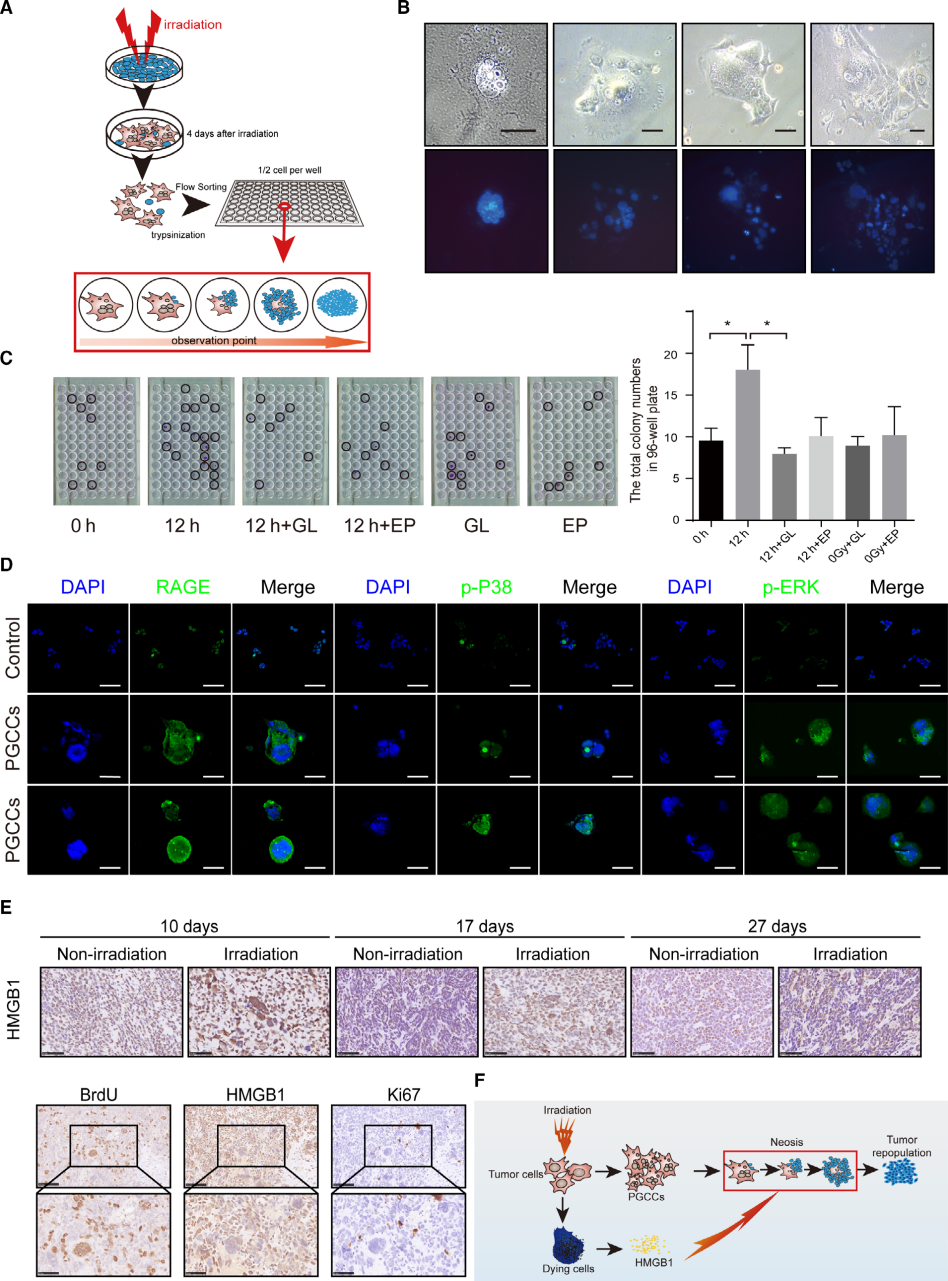
The HMGB1 secreted from dying cells promote neosis‐based tumor repopulation. (A) Schematic diagram showing the method of single‐cell cloning assay. (B) Upper panel, time‐lapse microscopy showing the process of neosis from one PGCC. Scale bar: 100 μm. Lower panel, Hoechst 33342 staining showing the process of neosis from one PGCC. Scale bar: 100 μm. (C) Black circles on 96‐well plates showing neosis‐initiating colonies from one single PGCC. Left panel, two HMGB1 inhibitors, ethyl pyruvate (EP, 9 mm) and glycyrrhizinate (GL, 200 μm), notably compromised single‐cell cloning ability of PGCCs in conditioned media from 4T1 cells 12 h after 10Gy irradiation. Right panel, quantification analysis showing single‐cell cloning ability of PGCCs under indicated conditions. (D) Immunofluorescent staining of RAGE, p‐P38 and p‐ERK in PGCCs. Scale bar: 500 μm. (E) Irradiation‐induced HMGB1 expression *in vivo*. Upper panel, immunohistochemical staining of HMGB1 in 4T1 tumor‐bearing animal. Lower panel, immunohistochemical colocalized staining of BrdU, HMGB1 and Ki67. (F) Schematic diagram showing the theory of PGCC‐started and neosis‐mediated tumor repopulation process after irradiation, in which HMGB1 participated. *n* = 3, data are shown as mean ± SD. One‐way ANOVA was used to determine statistical significance: *P < 0.05, **P < 0.01. [Colour figure can be viewed at wileyonlinelibrary.com]

HMGB1 is an important damage associated molecular pattern (DAMP) [30] that can be released from apoptotic cells [31] or necrotic cells [32]. Our previous studies revealed that HMGB1 from dying tumor cells stimulated the proliferation of living tumor cells and promoted tumor repopulation [33, 34]. In this study, we found that abundant HMGB1 was released during irradiation (Fig. 1C). We subsequently explored the possible role of HMGB1 in PGCC‐derived neosis. First, we utilized FACS to sort PGCCs formed 4 days after irradiation and used single‐cell cloning assay to observe the destiny of these PGCCs (Fig. 5A). This single‐cell cloning assay excluded the possibility that the newly formed colonies were an aggregate of many cells and the assay can also measure the neosis ability of PGCCs. As shown in Fig. 5B, some PGCCs indeed formed new cell colonies through cell budding (also called neosis). Since western blot analysis showed that HMGB1 in supernatant increased after irradiation (Fig. 1C), we then used the conditioned media from irradiated 4T1 cells to perform the single‐cell cloning assay. Fig. 5C showed that the conditioned media from irradiated 4T1 cells could significantly increase the colony formation of PGCC‐derived neosis. More importantly, two HMGB1 inhibitors, EP and GL, markedly neutralized the single‐cell cloning ability of PGCCs (Fig. 5C), suggesting that the HMGB1 in the conditioned media from irradiated 4T1 cells was involved in this process. Since our previous study reported that HMGB1 triggered a mitogenic signal in neighboring cells via the HMGB1‐RAGE‐P38/ERK signaling pathway [33], we stained PGCC with immunofluorescent RAGE, p‐P38 and p‐ERK. As shown in Fig. 5D, PGCCs presented stronger RAGE expression and a higher phosphorylation level of P38 and ERK compared with non‐irradiation cells. Additionally, we analyzed the expression of HMGB1 in the irradiation‐treated animal tumor bearing model. As shown in Fig. 5E, the expression level of HMGB1 increased after irradiation compared with non‐irradiated group. Moreover, some PGCCs presented high HMGB1 expression. Interestingly, we also observed some PGCCs that were BrdU‐positive but Ki67‐negative, or BrdU‐negative but Ki67‐positive, revealing that the proliferation potential of PGCCs varied; in other words, some PGCCs proliferated at an early stage and others at a late stage.

To summarize our findings:

- X‐ray irradiation could induce the formation of PGCCs, which could move towards either cell death and survival;
- irradiation‐generating PGCCs had proliferative potential and were involved in tumor cell repopulation after irradiation via neosis;
- HMGB1 released from dying cells stimulated the process of neosis and participated in tumor repopulation after irradiation (Fig. 5F).

## 4 Discussion

PGCCs can be observed in both tumors and tumor‐derived cell lines [8, 35]. Their expression was elevated after exposure to cancer therapy [10, 11, 12, 17, 19, 36]. Earlier reports found that PGCCs eventually die through mitotic catastrophe [37, 38, 39]; however, emerging studies have proved that PGCCs can form numerous small mononuclear cells, a process called neosis [20, 22, 24]. Also, increasing evidence shows that neosis deriving from PGCCs has a potential role in therapeutic resistance [23, 24], which may have been undervalued previously. Despite recent advances in the understanding of PGCC‐related cancer progression, the direct role of PGCC‐derived neosis in tumor repopulation or recurrence is poorly understood. In this study, we aimed mainly to illustrate the directly participatory role of neosis in tumor cell repopulation after radiotherapy. We first found that irradiation‐induced PGCCs could eventually die, in accordance with the findings of other studies [7, 10]. In spite of DNA damage by irradiation, some of the PGCCs still had proliferative ability and were observed to contribute to tumor repopulation through neosis. A previous study in our laboratory also revealed that HMGB1 from dying tumor cells stimulated the proliferation of living tumor cells and presaged poor prognosis in cancer patients, but did not examine the effect of HMGB1 on neosis and tumor repopulation after radiotherapy [34]. In contrast, the results of the present study demonstrated that HMGB1 released from dying tumor cells mediated the process of neosis and eventually bring about tumor repopulation (Fig. 5F).

Our results showed that the ratio of PGCCs following irradiation peaked at day 5 and decreased thereafter (Fig. 1E). The drop in the ratio of PGCCs in later days was attributed possibly to death of PGCCs, because some studies have reported the death of PGCCs [10, 37, 38, 39]. It is also highly possible that repopulation of newly emerging cells through neosis caused this ratio to drop as well, because we found that PGCCs contributed to tumor repopulation after radiotherapy via neosis (Figs 4A–E and 5B). The tumor colony was repopulated 15 days after irradiation, and the newly formed tumor cell repopulation still contained a higher percentage of PGCCs than in the non‐irradiated group (Fig. 1E), suggesting the non‐negligible role of PGCCs in tumor cell repopulation. Western blot analysis showed that Cleaved Caspase‐3 appeared after day 1 following irradiation, rose gradually in the following days, reached the peak at day 4 or 5, and decreased 6 days after irradiation (Fig. 1C). The trend of Cleaved Caspase‐3 expression following irradiation was consistent with that of the ratio of PGCCs after radiation (Fig. 1E,G), suggesting that PGCCs may also die through the process of apoptosis. As shown in Fig. 1G, a larger dose of irradiation induced a higher ratio of PGCCs (47.8% versus 16.1%, 14Gy versus 6Gy on day 3), but these PGCCs were more likely to die (10.6% versus 23.1%, 14Gy versus 6Gy on day 15), suggesting that PGCCs induced by a lower dose of irradiation survived for a longer period of time, due probably to less severe DNA damage.

We used BrdU pulse‐chase assay to confirm whether the PGCCs possessed proliferative ability (Fig. 2E). The result of this assay indicated that there was new formation of PGCCs and BrdU incorporated into DNA was attenuated with continuous cell division activity. The results do not support PGCCs as a resting or dormant cell, even though the emerging PGCC in 15 days after irradiation may be newly formed. In addition to this assay, we utilized multiple methods to uncover the proliferative capacity of some PGCCs (Figs 2D, F and 3D), with the unexpectedly finding that in spite of DNA damage, some PGCCs still had proliferative potential. To demonstrate clearly the fate of those proliferative PGCCs, we utilized time‐lapse microscopy to observe the phenomenon of neosis (Figs 4A–D and 5B). Fortunately, we precisely captured the process of neosis, in which two small Raju cells are being expelled from the PGCC (Fig. 4C). We observed that the RCs had stronger proliferative ability and a lower level of apoptosis (Fig. 4E), which was consistent with the opinion that neosis mediates tumor progression after therapy [20, 21, 22] and therapy resistance [23, 24]. We applied a soft agar assay to test the long‐term clonogenic potential of PGCCs and found that irradiation‐induced PGCCs had the ability to form spheroids (Fig. 4F). Moreover, these cells had a higher stemness potential (Fig. 4G), consistent with previous studies [7, 25, 40]. To measure quantitatively the role of PGCC‐derived neosis in tumor repopulation after radiotherapy, we innovatively modified the single‐cell cloning assay (Fig. 5A,B). We also explored the potential role of HMGB1 in neosis‐based tumor repopulation (Fig. 5C). Intriguingly, our results showed that HMGB1 in the extracellular space promoted PGCC‐derived neosis in tumor repopulation (Fig. 5C). Furthermore, we found that the expression of RAGE, p‐P38 and p‐ERK was elevated in PGCCs and RCs (Fig. 5D), implying that HMGB1/RAGE/P38 and ERK might participate in this process.

This study reveals for the first time the role of HMGB1 in neosis‐based tumor repopulation, consolidating and enriching the role of HMGB1 in tumor recurrence after therapy [41] and tumor progression [42, 43].

## 5 Conclusion

Our findings demonstrate that irradiation‐induced PGCCs contribute to tumor repopulation via neosis, in which HMGB1 is involved. We hope that the theory of PGCC‐derived neosis on tumor repopulation we propose in this study can provide novel insights to understand the process of tumor repopulation after therapy at cellular and molecular levels.

## Ethics approval and consent to participate

The animal study was approved by the Animal Ethics Committee and Ethical Review Board of Shanghai General Hospital, Shanghai Jiao Tong University School of Medicine, China.

## Consent for publication

All authors agree to publication.

## Data availability

The authors declare that all data supporting the findings of this study are available within the article and its additional files, or through contact with the corresponding author upon reasonable request.

## Conflict of interest

The authors declare that they have no competing interests.

## Author contributions

ZZ, SH, QH: designed the study and interpreted data and approved the manuscript before submission. SH, XF: contributed to manuscript writing and approved the manuscript before submission. ZD, JC, YW, MZ, YZ: collected and analyzed data and approved the manuscript before submission. JC, QH: gave constructive comments on the manuscript and approved the manuscript before submission.

## Supporting information


**Fig. S1.** Cisplatin induced the formation of PGCCs. White star showed the PGCC and yellow star showed Raju cell. Scale bar: 50__m.
