# Supplementary figures and images for: Irradiation‐induced polyploid giant cancer cells are involved in tumor cell repopulation via neosis
Source: Mol Oncol. 2021 Feb 17;15(8):2219–34. doi: 10.1002/1878-0261.12913 (PMC8334289; doi:10.1002/1878-0261.12913)

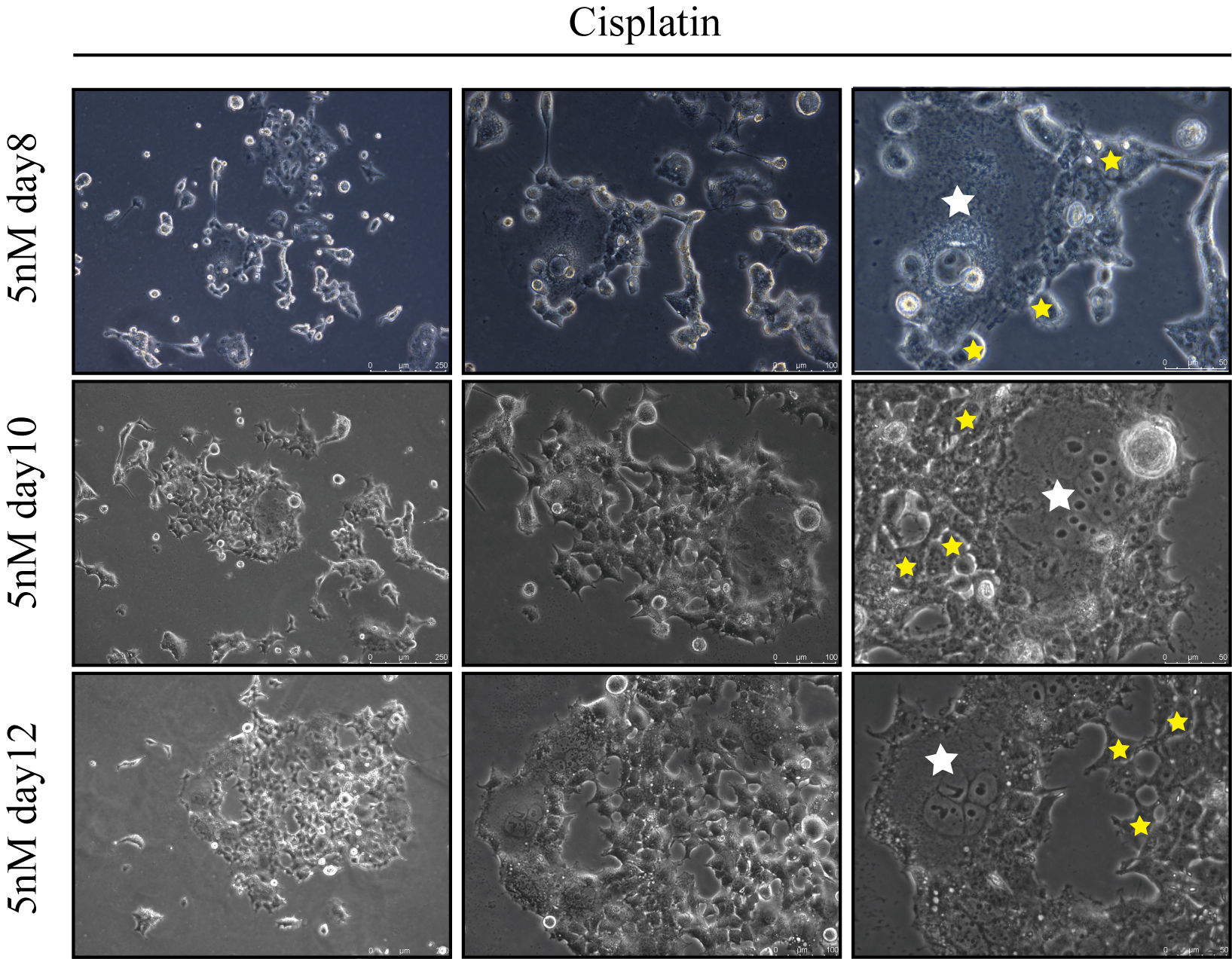

Supplement: Supplementary file 1 — Fig. S1. Cisplatin induced the formation of PGCCs. White star showed the PGCC and yellow star showed Raju cell. Scale bar: 50__m. [file MOL2-15-2219-s001.jpg]
